# Vestibular-Dependent Functions Following MRgLITT—A Single-Group Longitudinal Study

**DOI:** 10.3390/brainsci15020181

**Published:** 2025-02-12

**Authors:** Milos Dordevic, Kiana Assady Looyeh, Friedhelm C. Schmitt, Notger Müller

**Affiliations:** 1 Degenerative and Chronic Diseases of the Faculty of Health Sciences (FGW), University Potsdam, 14469 Potsdam, Brandenburg, Germany; 2 Department of Neurology, Otto-von-Guericke-University Magdeburg, 39106 Magdeburg, Sachsen-Anhalt, Germany

**Keywords:** vestibular, epilepsy, TLE, MRgLITT, laser surgery

## Abstract

Background: Temporal lobe epilepsy is the most common pharmaco-resistant type of epilepsy. The chance of obtaining seizure freedom after resective surgery in pharmaco-resistant mesial temporal lobe patients (mTLE) is significantly higher compared to pharmaceutical treatment (at least 50–60% compared to less than 15%). However, some factors (e.g., craniotomy) may prevent epilepsy patients undergoing surgery. A recent advancement in epilepsy surgery, i.e., magnetic resonance guided laser interstitial thermal therapy (MRgLITT), has become an attractive alternative for performance of selective amygdala-hippo-campectomy, especially because of its minimal invasiveness. Among other medial temporal lobe structures, the hippocampus is particularly important for successful processing of vestibular inputs. Nevertheless, it is still unclear whether mTLE patients who underwent MRgLITT perform worse on vestibular-dependent tests, including balancing, spatial orientation and rotational memory. Methods: Nine patients (Age 40.1 ± 14.5; 2 females) underwent vestibular-dependent assessments before and after MRgLITT using the following test battery: (I) clinical balancing test (CBT), (II) triangle completion test (TCT) and (III) rotational memory test (RM). Results: We found significant improvement from pre- to post-surgery in the vestibular-dependent spatial orientation test, namely in the wheelchair condition of the triangle completion test. Additionally, the obtained effect sizes were medium to large in favor of post-surgery assessment for the majority of conditions in the three tests applied in this study, indicating that the assessment of a larger number of patients could also, potentially, lead to significant results in these cases. Conclusions: This plausibility study is the first to assess vestibular-dependent balancing, spatial orientation and rotational memory functions before and after MRgLITT in mTLE patients. Even with a small sample of nine patients, significant changes and medium to high effect sizes in favor of surgery were observed. Nevertheless, prospective studies with larger sample sizes are necessary for appropriate estimation of MRgLITT effectiveness in these functional domains.

## 1. Introduction

Epilepsy affects over 50 million people worldwide, with temporal lobe epilepsy (TLE), originating primarily from the hippocampus, entorhinal cortex, amygdala and temporal pole, representing the most common drug-resistant epilepsy in adults [1].

Besides structural brain alterations, such as atrophy [2,3], numerous functional and cognitive consequences of TLE have been reported, both in animal models and humans. For instance, earlier studies found deficits in spatial learning and memory in the Barnes maze test in mouse models of TLE [4]. Likewise, performance of rats on hippocampus-dependent spatial tasks was altered after pilocarpine-induced seizures, with deficits remaining throughout the chronic stage, which correlated with theta oscillations [5]. Perhaps the most well-known human case of memory deficits following bilateral amygdala-hippo-campectomy is patient H.M. (Henry Molaison 1926–2008), for whom the disruption of spatial learning and memory has been studied most intensively [6]. Deficits in determining one’s own position with closed eyes, by monitoring internally generated self-motion signals (called path integration), were reported in TLE patients who had undergone anterior temporal lobe (aTL) resection, on tasks assessing both linear and rotational locomotion [7,8,9]. In addition, some studies reported balance deficits in TLE patients [10], as well as neurobiological alterations in brain regions known to process vestibular information [11]. In our recently published work [12] we found deficits in vestibulo-hippocampal functions, i.e., spatial orientation and path integration, rotational memory and balancing, in a group of twenty operated and non-operated TLE patients.

In comparison to temporal lobe resection, MRI-guided laser interstitial thermal therapy (MRgLITT) is minimally invasive and, as such, constitutes a promising approach for a safe and effective treatment of TLE patients [13,14,15]. However, previous studies reported disparate effects of this temporal lobe laser surgery: while improvements could be seen in object naming and recognition [16] and verbal memory [17], various rates of decline were found for attention, motor cognition and general executive functions [13]. The impact of this relatively novel surgical method on vestibular-dependent functions, however, remains unknown. Therefore, in this longitudinal study, we assessed nine MTL epilepsy patients before and after MRgLITT using the same test battery as in our previous study [12]: (I) spatial orientation and path integration, (II) rotational memory and (III) balancing abilities. We hypothesized improvements in these functions after surgery, arising from the eliminated negative influence of epileptic seizures in brain regions responsible for processing respective inputs.

## 2. Materials and Methods

### 2.1. Participants and Study Design

In total, twenty TLE patients (37.0 ± 15.1 years, two females) were admitted to this study based on inclusion criteria (Table 1), through the Epilepsy Department of the Otto von Guericke University Clinic for Neurology in Magdeburg. The age range of TLE patients in this study was from 18 to 80 years. The epileptogenic zone [18] was determined by video electroencephalography (EEG) monitoring (i.e., seizure semiology and ictal EEG) and neuroimaging evaluation (MRI and positron emission tomography (PET)). Typical epileptogenic lesions detected by MRI were hippocampal sclerosis or WHO Grad 1 tumors. No patient had subjective symptoms or clinical signs of a cerebellar syndrome, typically seen in patients with drug-induced vestibular dysfunction. All patients had focal onset seizures with impaired awareness (semio-logically auto-motor seizures); most had focal onset aware seizures (auras) and rare focal to bilateral tonic–clonic seizures, typical for MTLE, and nine patients underwent a MRgLITT surgery (Table 1).

This study was prospective, longitudinal and single-blinded (analysis) with one factor: time (pre, post). All assessments took place in the German Center for Neurodegenerative Diseases (DZNE) from June 2021 to June 2023. A brief overview of the main characteristics of patients is shown in the table above (Table 1).

### 2.2. MRgLITT Surgery

**Figure 1:**
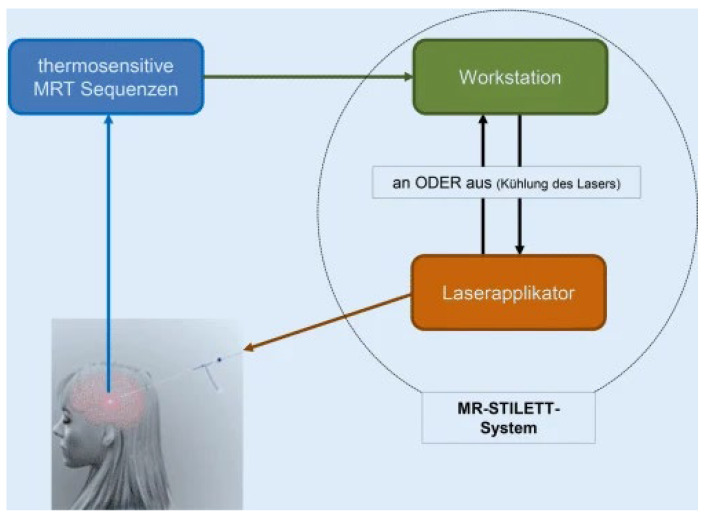
Schematic presentation of the loop between stereotactic laser thermo-ablation system, laser applicator (orange) and thermosensitive MRI-sequence (blue); on/off for laser cooling is presented bidirectionally between the workstation (green) and the laser applicator (orange) [19].

The stereo-tactically guided laser ablation system (Visualase-Thermal-Therapy System^®^, Medtronic, Minneapolis, MN, USA) was approved by the FDA in 2007. The first application with epilepsy patients was performed by Curry et al. in 2011. Technically, it combines a 15 W, 980 nm diode laser and a cooled laser application system with a computer station for image processing. The MR-compatible laser applicator measures 1.6 mm and contains a central 400 μm silicon core in the fiber optic applicator with a light-scattering tip (outer diameter 0.76 mm) and an outer flexible translucent cooling shaft. The applicator causes cylindrical ellipsoidal light distribution over a distance of either 3 or 10 mm. To monitor thermal therapies, a “thermal dose model”, based on the Arrhenius damage integral, is used: this quantifies damage by including temperature and time in a non-linear way. In the case of the Visualase^®^ system, the ablation area is in relation to an equivalent heat time at 43 °C. In biological tissue at this temperature, a thermal dose of 25–240 min is required to induce total tissue necrosis. The Visualase^®^ system allows adjustment of the energy output of the laser to the desired lesion radius, so that only a few minutes are required for the actual lesioning. The higher energy output means, however, that the penetration depth decreases in comparison. The workstation enables a near real-time calculation (5 s) of the ablation zone. Taking into account the ratio of the shift of the proton resonance frequency [3], the shift of the image phase is linearly correlated with the temperature change. Based on the calculated temperature values, the computer station provides color-coded temperature maps. Time and temperature curve data are recorded for each voxel. The Arrhenius model of thermal destruction then enables use of this information prediction for an irreversible damage zone. During the procedure, the applicator is connected to a peristaltic roller pump. This circulates sterile solution through the cooling shaft to the laser fibers, as well as the surrounding tissue, thus preventing tissue carbonization. To increase procedural safety, the software, before the ablation, allows definition of temperature maxima in singular voxels. If these maxima are exceeded. the laser is automatically switched off (see Figure 1). For details on the surgical procedure, see [19].

The CE-certification of this treatment option was issued in 2018 in Europe for one of the two US certified systems: the Visualase System. The procedure was carried out at the Otto-von-Guericke University Clinic for Neurosurgery in Magdeburg. Open microsurgical resection and stereotactic laser thermal ablation were discussed in detail with the selected patients. Patients agreed to undergo this procedure because of lower invasiveness, as well as the continued possibility of being able to resort to a resective procedure if required (i.e., a step-by-step procedure). High-resolution MRI, functional MRI and positron emission tomography and simulated brachytherapy were used in preoperative planning, as part of the stereotactic planning procedure related to the ablation volume. The imaging data were used to determine the exact position and size of the epileptogenic focus or target volume for ablation. In addition, the optimal access route and the exact placement of the laser fiber were calculated, so the risk of damaging neighboring healthy tissue would be minimized. The stereo-tactical information for preoperative planning was transferred in the surgical setting. Intraoperatively, a fiber-optic laser probe with a diameter of less than one millimeter was inserted and fixed via a small hole in the skull, under general anesthesia. During surgery, real-time MRI (with a delay of approximately 3–4 s) was used to track the position of the laser probe, as well as to enable continuous adjustment of the energy output and the position of the laser probe. Usually two ablation sites within one trajectory was sufficient: the ablations resulted in a typical sandglass formation involving almost the complete AHC. Intra-operatively, a morphological MRI was performed to compare the actual ablation volume with that in preoperative planning. Post-operatively, another MRI was performed to evaluate the ablated volume and to exclude unexpected side effects, such as extensive operative edema, bleeding or accidental ablation of other brain structures.

### 2.3. Tests

All tests were performed within one week before (pre-test) and after (post-test) surgery.

#### 2.3.1. Clinical Balance Test (CBT)

The clinical balance test contained standing, on stable and unstable surfaces, and walking conditions. Furthermore, both contain sub-conditions with open and closed eyes. In total, the maximal number of points that could be collected by participants is 90, with each of 30 assessment items carrying a minimum of 0 and a maximum of 3 points For further details on this test, please refer to our previously published work [12,20,21].

#### 2.3.2. Triangle Completion Test (TCT)

The triangle completion test (TCT) assesses participant’s non-visual spatial orientation abilities. In this test, six triangular paths were marked on the floor of a room, three going towards the left and three going towards the right, thus creating three pairs of triangular paths. The turning angles for these three triangular paths were 60°, 90°, and 120°. Each participant was tested under two conditions: walking (active) and being pushed in a wheelchair (passive). Each of these conditions applied along each of the paths (60°, 90°, and 120°) Overall, this resulted in 12 trials per participant (three to the left and three to the right, multiplied by the two conditions). After each run, participants were asked to walk directly back to the starting point using the shortest possible route. The main outcome variables were the distance and the angular error. Additional information on this test can be found in [12,20,21].

#### 2.3.3. Rotational Memory (RM)

During the rotational memory test, as in our previous studies [12,21], each subject was seated in a chair that rotated (Interacoustics, Middelfart, Denmark) in the transverse plane (left and right rotations about a vertical axis). Participants received eye masks and earmuffs, so they would be blindfolded and could hear no sound for the duration of the test. Initially, software executed predetermined rotations of the chair. Following this, participants were asked to estimate the current location in relation to the original location. In relation to this, participants would request either to remain at their current location (if considered to be the same as the original location) or to be rotated (left or right) towards the original location. The following rotations were executed twice each by the software: one, two, four, and eight rotations. After each trial, the chair was automatically rotated back to the initial position. The variable of interest was angular error, determined as angular distance between the original location and the location estimated to be the original one by the patient.

### 2.4. Statistical Analysis

All statistical analysis were performed with SPSS v.21 (IBM, Armonk, NY, USA). Before running statistical tests, all data were first checked for assumptions of normality and homogeneity of variance. Dependent *t*-test or Wilcoxon test (when assumptions were not fulfilled) were used to investigate differences between pre- and post-assessments. The table shows respective means and standard deviations, as well as effect sizes (Cohen’s d). In the figures, respective means, with 95% confidence interval of the difference between means, are depicted.

## 3. Results

Complete datasets of nine MRgLITT-operated patients from CBT and TCT were obtained and analyzed. Two patients could not be tested on RM at the post-test, due to technical issues.

**Figure 2:**
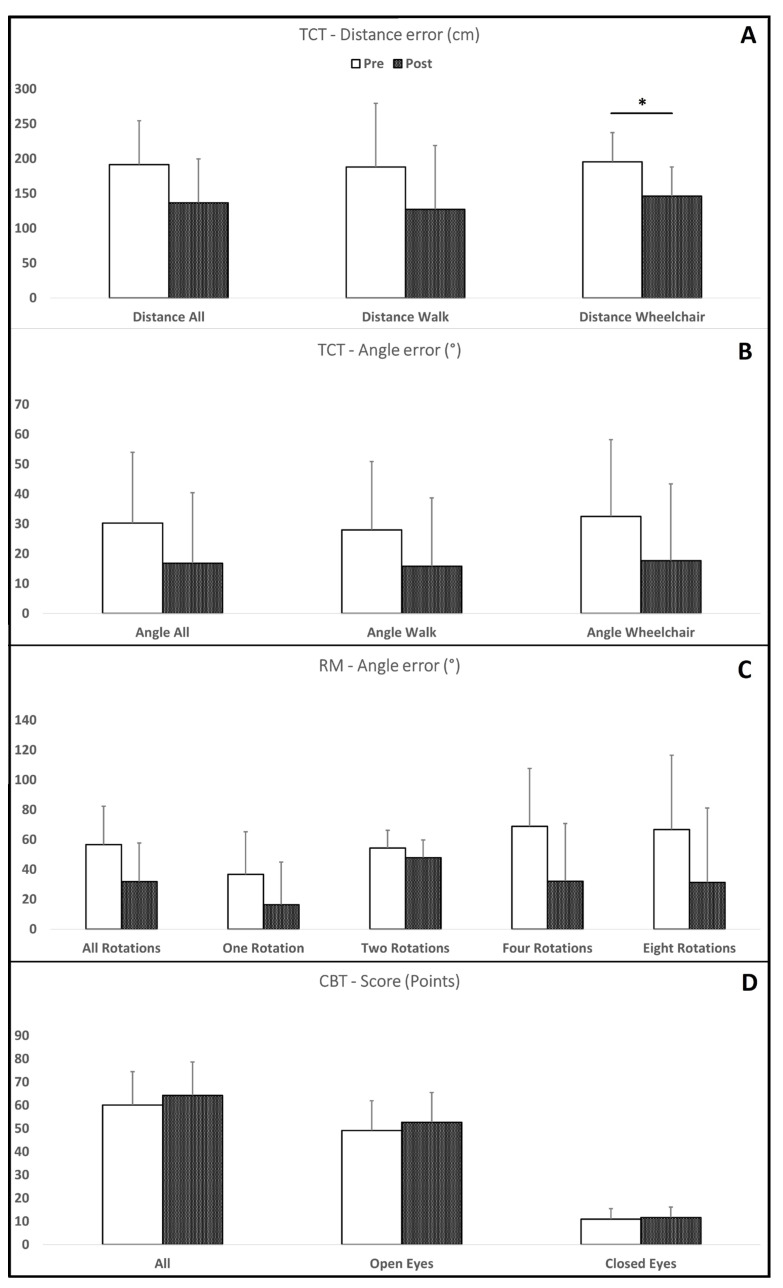
Errors on TCT (**A**,**B**), RM (**C**) and score on CBT (**D**) for all conditions for both pre- and post-test; * *p* < 0.05; TCT—triangle completion test, RM—rotational memory test, CBT—clinical balance test.

The patients performed significantly better for the vestibular-dependent (wheelchair) condition of the TCT at post-test compared with pre-test (Figure 2A, Table 2), where they demonstrated decreased ability to return to the starting point, represented by a larger distance from the point where they ended up, relative to the original starting point. Although other tests and conditions did not reach statistical significance (Figure 2B–D, Table 2), a very large effect size was found for the overall rotational memory test and large or medium effect sizes for all other tests and conditions (Table 2). Further sub-group analyses were not performed because of the relatively small sample size.

## 4. Discussion

The results partly confirmed our hypothesis, with significantly better performance following MRgLITT on the vestibular-dependent spatial orientation test (wheelchair condition for TCT). Other tests revealed non-significant results; however, the obtained effect sizes on all tests and conditions ranged from medium to very strong in favor of MRgLITT, indicating that a larger sample of patients might have led to more significant results.

Epilepsy surgery remains the main means of therapy for patients with drug-resistant TLE [22], wherein MRI-guided laser interstitial thermal therapy (MRgLITT) is among the most promising approaches [23], with around half of patients achieving long-term Engel I seizure freedom [15], especially when ablations were performed along a more anterior, medial, and inferior trajectory [24]. Interestingly, in this large multicenter study with 234 patients, the occurrence of postoperative complication was associated with poorer seizure outcome, so that a suboptimal trajectory may also result in an increased likelihood of complications. Complications attributable to the procedure itself were visual disturbances, such as double vision or partial visual field defect (5.1%), and postoperative hemorrhage (1.3%), In our cohort, only transient double vision was observed one time.

The most affected brain regions in TLE pathology are the hippocampus, entorhinal cortex, amygdala and temporal pole; in these regions, atrophy and interhemispheric asymmetry may also be detected [2]. More specifically, studies suggested a reorganization of lateralization patterns in hippocampal activity for the visual–spatial task, e.g., patients with non-dominant mesial TLE due to hippocampal sclerosis reveal shifts in brain activity to the left hemisphere, interpreted as an adaptive memory reorganization in the healthy hemisphere [25]. Interestingly, a shift to the contralateral superior temporal gyrus could also be detected after non-dominant SAHE following MRgLITT in a small cohort of nine patients. Results of one study revealed a relatively large decline in ipsilateral mammillary body volume in TLE patients who underwent successful SAHE via MRgLITT, when compared to patients without seizure control after the same intervention [26]. Bilateral mammillary damage causes anterograde amnesia in patients, so one could expect in the seizure-free group a decline in non-verbal memory function. Unfortunately, the authors did not provide any data for this question. The Houston group repeatedly reported a higher rate of verbal memory impairment, especially following language-dominant resective surgeries. Others even found that an improvement in general, functions, such as naming or object recognition, may be more preserved following MRgLITT compared to open surgical procedures, possibly due to the lack of “collateral damage” to the lateral temporal regions outside the hippocampus. Moreover, although the hippocampus has long been considered an important structure supporting episodic and declarative memory, neuropsychological assessments following epilepsy surgeries suggest that declarative memory deficits are greater following lesions in the para-hippocampal gyrus and in the lateral TL [13,16,27,28].

Our previously published work revealed deficits in TLE patients, when compared to healthy controls, on all tests used in this study [12]. However, the lack of an interventional design did not allow us to draw causal conclusions on why TLE patients show deficits in vestibulo-hippocampal spatial abilities. In addition, the patients group consisted both of non-operated TLE patients, whereby less than half of patients underwent open surgical intervention (temporal lobectomy) and none underwent MRgLITT. Nevertheless, up to the point of writing this paper, we were unable to find similar studies investigating spatial orientation abilities in TLE patients before and after MRgLITT, which prevents us from directly discussing the results obtained. Still, it is known from animal models that TLE might cause a lower ability in spatial searching of optimal paths in a maze (4); some TLE animal models, with hippocampus-based epilepsy, revealed spatial memory deficits, with non-spatial memory remaining unaffected, related to theta oscillation mechanisms [5]. It has also been proposed in humans that the right hemisphere plays a dominant role in these abilities; for instance, right but not left temporal lobectomy leads to deficits in linear path integration, as measured by patients’ ability to estimate their own linear displacement while walking to previously seen targets at 2 to 6 m [7]. Similar findings were reported when temporal lobectomy patients’ ability was assessed on the triangle completion task, with only right lobectomy patients showing deficits, suggesting that the right temporal lobe plays a role in spatial memory based on internal cues [8]. Allocentric navigation in TLE patients was found to be impaired irrespective of epilepsy lateralization, with good and poor navigators not differing in their age, gender, or preoperative/postoperative status [29]. It remains, however, unclear why patients performed better following MRgLITT on the vestibular-dependent spatial orientation test in this study. Potential learning effects can be excluded, because we assessed them in our earlier studies. Given the preciseness of MRgLITT, one may speculate that this surgical procedure leads to removal of seizure-caused functional disturbances from temporal brain areas crucial for this ability, without damaging the lateral temporal areas, as occurs in resective surgery. However, this explanation remains at a speculatory level, with future studies required on larger and matched samples of patients with control groups. More importantly, a better understanding of the functional nature and the anatomical allocation of the network which plays the essential role for the vestibular-dependent spatial orientation is essential. Additional tests, such as MEG, EEG, MRI and especially fMRI, may be needed to reach this goal. Indeed, it has been shown that ictal symptoms are usually related to a discharge involving temporal or parietal areas, which are supposed to be a crucial component of the so-called “vestibular cortex”. The ictal EEG/fMRI can reveal the main activation clusters in the temporo-parieto-occipital regions, which are known to be involved in the processing of vestibular information [11]. Previous studies, including our own, have shown that TLE patients show worse balancing skills compared to healthy controls [10,12]. In this study, there was no significant difference pre- to post-surgery in this ability, which might imply that MRgLITT affects mainly higher level vestibulo-hippocampal functions, such as vestibular-dependent spatial orientation.

This study contains a number of limitations. Due to its feasibility nature, we were unable to provide adequate control participants, which represents one of the major limitations with regards to drawing conclusions. In a planned future larger scale interventional study, this will be accounted for. Another major limitation is the relatively small sample size of participants; however, it is meaningful to run a plausibility study on a small number of participants before organizing a larger scale study, which requires significantly more resources. In addition, although visual input was not crucial for the majority of vestibular-dependent tasks in this study, it had to be used to, e.g., memorize target locations and, therefore, a non-visual task should have been used in parallel for obtaining results. Although visual field deficits in TLE patients can have an effect on the study results, they are more common in patients treated with open temporal lobectomy compared to laser-based minimally invasive procedures [26,30]. It is also of importance to mention that doses of some medications are known to affect performance on spatial memory tasks, which could have also been the case in our study [31]. In summary, a cohort which is matched to the seizure duration, language lateralization and drug load would be ideal. Besides assessments of non-verbal memory, psychological factors, such as fatigue and stress, before and after the testing, as well as additional information about patients’ well-being (e.g., quality of life and depression scale) should be also taken into account in future studies. Complementary imaging assessments (i.e., MRI) are also recommended, in order to make more thorough conclusions about relevant findings.

## 5. Conclusions

In conclusion, this study revealed that MRgLITT-patients perform better on a vestibulo-hippocampal spatial orientation test, i.e., the wheelchair condition of the triangle completion test, following surgery, compared to the pre-surgery level. Although not reaching significance, on most other spatial orientation conditions, strong effect sizes could also be seen following MRgLITT, except on the clinical balancing test, where patients performed similarly to the pre-surgery level. These results need to be confirmed or discredited in future studies on larger and matched samples of patients and in a design containing control groups and possibly healthy volunteers.

## Figures and Tables

**Table 1:** Characteristics of patients.

Patient (P)	Age (y)	Gender	Time Since First Seizure (y)	Language Laterality	No. of AEDs at Time of the Study	Education (y)	MTL Sclerosis
P1	19	M	15	Right	2	9	Right
P2	61	F	44	Left	3	13	Left
P3	54	F	21	Bilateral (Left > Right)	5	9	/
P4	36	M	20	Right	1	15	/
P5	30	M	29	Right	3	13	Right
P6	35	M	29	Right	4	12	Right
P7	31	M	3	Right	3	9	/
P8	30	F	24	Right	3	17	Right
P9	17	M	2	Left	3	15	/

**Table 2:** Results of all tests and conditions; SD—standard deviation, *—*p* < 0.05, §—d > 0.8.

Test	Condition(s)		Mean ± SD Pretest	Mean ± SD Posttest	95% CI	*p*-Value	Effect Size (d)
Triangle Completion Test (TCT)	Angle	All Conditions	30.3 ± 27.7	16.8 ± 5.6	23.7	0.217	0.48
		Walk	28.0 ± 26.3	15.8 ± 8.9	22.9	0.247	0.44
		Wheelchair	32.5 ± 30.5	17.7 ± 3.5	25.7	0.219	0.49
	Distance	All Conditions	191.6 ± 119.1	136.7 ± 56.2	62.9	0.079	0.67
		Walk	188.0 ± 163.6	127.2 ± 69.6	91.6	0.165	0.49
		Wheelchair	195.5 ± 80.7	146.1 ± 45.7	41.9	0.027 *	0.90 ^§^
Rotational Memory (RM)	All Conditions		56.6 ± 36.2	31.9 ± 17.2	25.8	0.059	0.89 ^§^
	One Rotation		36.7 ± 29.1	16.4 ± 7.2	28.6	0.132	0.66
	Two Rotations		54.3 ± 33.8	47.9 ± 34.1	11.9	0.233	0.49
	Four Rotations		68.9 ± 46.9	32.1 ± 24.7	38.7	0.059	0.88 ^§^
	Eight Rotations		66.7 ± 57.6	31.4 ± 31.1	49.8	0.134	0.66
Clinical Balance Test (CBT)	All Conditions		60.1 ± 12.2	64.3 ± 15.1	14.4	0.116	0.59
	Open Eyes		49.1 ± 10.5	52.7 ± 13.3	12.8	0.134	0.55
	Closed Eyes		11.0 ± 2.6	11.7 ± 2.3	4.5	0.397	0.30

## Data Availability

Data are available only under special permission due to data protection regulations.

## References

[1] Thijs R.D., Surges R., O’Brien T.J., Sander J.W. (2019). Epilepsy in adults. Lancet.

[2] Larivière S., Royer J., Rodríguez-Cruces R., Paquola C., Caligiuri M.E., Gambardella A., Concha L., Keller S.S., Cendes F., Yasuda C.L. (2022). Structural network alterations in focal and generalized epilepsy assessed in a worldwide ENIGMA study follow axes of epilepsy risk gene expression. Nat. Commun..

[3] Park B.-Y., Larivière S., Rodríguez-Cruces R., Royer J., Tavakol S., Wang Y., Caciagli L., Caligiuri M.E., Gambardella A., Concha L. (2021). Topographic divergence of atypical cortical asymmetry and atrophy patterns in temporal lobe epilepsy. Brain.

[4] Herrewegen Y.V.D., Denewet L., Buckinx A., Albertini G., Van Eeckhaut A., Smolders I., De Bundel D. (2018). The Barnes Maze Task Reveals Specific Impairment of Spatial Learning Strategy in the Intrahippocampal Kainic Acid Model for Temporal Lobe Epilepsy. Neurochem. Res..

[5] Chauvière L., Rafrafi N., Thinus-Blanc C., Bartolomei F., Esclapez M., Bernard C. (2009). Early deficits in spatial memory and theta rhythm in experimental temporal lobe epilepsy. J. Neurosci..

[6] Murphy G.G. (2013). Spatial learning and memory—What’s tle got to do with it?. Epilepsy Curr..

[7] Philbeck J.W., Behrmann M., Levy L., Potolicchio S.J., Caputy A.J. (2004). Path integration deficits during linear locomotion after human medial temporal lobectomy. J. Cogn. Neurosci..

[8] Worsley C.L., Recce M., Spiers H.J., Marley J., Polkey C.E., Morris R.G. (2001). Path integration following temporal lobectomy in humans. Neuropsychologia.

[9] Yamamoto N., Philbeck J.W., Woods A.J., Gajewski D.A., Arthur J.C., Potolicchio S.J., Levy L., Caputy A.J. (2014). Medial temporal lobe roles in human path integration. PLoS ONE.

[10] Gandelman-Marton R., Arlazoroff A., Dvir Z. (2006). Balance performance in adult epilepsy patients. Seizure.

[11] Morano A., Carnì M., Casciato S., Vaudano A.E., Fattouch J., Fanella M., Albini M., Basili L.M., Lucignani G., Scapeccia M. (2017). Ictal EEG/fMRI study of vertiginous seizures. Epilepsy Behav..

[12] Dordevic M., Gruber J., Schmitt F.C., Mueller N. (2020). Impairments in path integration, rotational memory and balancing in patients with temporal lobe epilepsy. BMJ Neurol. Open.

[13] Brenner D.A., Valdivia D.J., Dadario N.B., Aiyathurai J., Mashiach E., Ginalis E.E., Quinoa T.R., Wong T., Sun H. (2024). Functional outcomes in MRI-guided laser interstitial therapy for temporal lobe epilepsy: A systematic review and meta-analysis. J. Neurosurg..

[14] Kerezoudis P., Parisi V., Marsh W.R., Kaufman T.J., Lehman V.T., Worrell G.A., Miller K.J., Van Gompel J.J. (2020). Surgical Outcomes of Laser Interstitial Thermal Therapy for Temporal Lobe Epilepsy: Systematic Review and Meta-analysis. World Neurosurg..

[15] Youngerman B.E., Banu M.A., Khan F., McKhann G.M., Schevon C.A., Jagid J.R., Cajigas I., Theodotou C.B., Ko A., Buckley R. (2023). Long-term outcomes of mesial temporal laser interstitial thermal therapy for drug-resistant epilepsy and subsequent surgery for seizure recurrence: A multi-centre cohort study. J. Neurol. Neurosurg. Psychiatry.

[16] Drane D.L., Loring D.W., Voets N.L., Price M., Ojemann J.G., Willie J.T., Saindane A.M., Phatak V., Ivanisevic M., Millis S. (2015). Better object recognition and naming outcome withMRI-guided stereotactic laser amygdalohippocampotomy for temporal lobe epilepsy. Epilepsia.

[17] Drane D.L., Willie J.T., Pedersen N.P., Qiu D., Voets N.L., Millis S.R., Soares B.P., Saindane A.M., Hu R., Kim M.S. (2021). Superior Verbal Memory Outcome After Stereotactic Laser Amygdalohippocampotomy. Front. Neurol..

[18] Rosenow F., Klein K.M., Hamer H.M. (2015). Non-invasive EEG evaluation in epilepsy diagnosis. Expert Rev. Neurother..

[19] Schmitt F.C., Büntjen L., Schütze H., Kaufmann J., Heinze H.-J., Hinrichs H., Tempelmann C., Düzel E., Voges J. (2020). Stereotactic laser thermal ablation of mesial temporal lobe epilepsy with right hippocampal sclerosis—Patient decision-making, realization and visualization of memory function. Z. Epileptol..

[20] Dordevic M., Hökelmann A., Müller P., Rehfeld K., Müller N.G. (2017). Improvements in Orientation and Balancing Abilities in Response to One Month of Intensive Slackline-Training. A Randomized Controlled Feasibility Study. Front. Hum. Neurosci..

[21] Dordevic M., Sulzer S., Barche D., Dieterich M., Arens C., Müller N.G. (2021). Chronic, Mild Vestibulopathy Leads to Deficits in Spatial Tasks That Rely on Vestibular Input While Leaving Other Cognitive Functions and Brain Volumes Intact. Life.

[22] Schmitt F.C., Meencke H. (2020). Factors predicting 10-year seizure freedom after temporal lobe resection: A monocentric, continuous extra-long-term evaluation. Z. Epileptol..

[23] Issa N.P., Warnke P. (2023). Interstitial laser ablation for epilepsy: Beauty lies in the eye of the beholder. J. Neurol. Neurosurg. Psychiatry.

[24] Wu C., Jermakowicz W.J., Chakravorti S., Cajigas I., Sharan A.D., Jagid J.R., Matias C.M., Sperling M.R., Buckley R., Ko A. (2019). Effects of surgical targeting in laser interstitial thermal therapy for mesial temporal lobe epilepsy: A multicenter study of 234 patients. Epilepsia.

[25] Sideman N., He X., Kim N.Y., Sperling M., Sharan A. (2016). Lateralization-of-Visual-Spatial-Memory-in-Temporal-Lobe-Epilepsy-Displays-Adaptive-Reorganization. https://aesnet.org/abstractslisting/lateralization-of-visual-spatial-memory-in-temporal-lobe-epilepsy-displays-adaptive-reorganization.

[26] Grewal S.S., Gupta V., Vibhute P., Shih J.J., Tatum W.O., Wharen R.E. (2018). Mammillary body changes and seizure outcome after laser interstitial thermal therapy of the mesial temporal lobe. Epilepsy Res..

[27] Kohlhase K., Zöllner J.P., Tandon N., Strzelczyk A., Rosenow F. (2021). Comparison of minimally invasive and traditional surgical approaches for refractory mesial temporal lobe epilepsy: A systematic review and meta-analysis of outcomes. Epilepsia.

[28] Drane D.L. (2018). MRI-Guided stereotactic laser ablation for epilepsy surgery: Promising preliminary results for cognitive outcome. Epilepsy Res..

[29] Amlerova J., Laczo J., Vlcek K., Javurkova A., Andel R., Marusic P. (2013). Risk factors for spatial memory impairment in patients with temporal lobe epilepsy. Epilepsy Behav..

[30] Voets N.L., Alvarez I., Qiu D., Leatherday C., Willie J.T., Sotiropoulos S., Gleichgerrcht E., Bonilha L., Pedersen N.P., Kadom N. (2019). Mechanisms and Risk Factors Contributing to Visual Field Deficits following Stereotactic Laser Amygdalohippocampotomy. Ster. Funct. Neurosurg..

[31] Wandschneider B., Stretton J., Sidhu M., Centeno M., Kozák L.R., Symms M., Thompson P.J., Duncan J.S., Koepp M.J. (2014). Levetiracetam reduces abnormal network activations in temporal lobe epilepsy. Neurology.

